# A G-Protein Coupled Receptor and Macular Degeneration

**DOI:** 10.3390/cells9040910

**Published:** 2020-04-08

**Authors:** Anna G. Figueroa, Brian S. McKay

**Affiliations:** Department of Ophthalmology and Vision Science, University of Arizona, Tucson, AZ 85724, USA; afigueroa@email.arizona.edu

**Keywords:** GPR143, melanin, RPE, G-protein, OA1, L-DOPA, macular degeneration, AMD

## Abstract

Age-related macular degeneration (AMD) is a leading cause of irreversible blindness in the world. The risk of AMD increases with age and is most common among the white population. Here, we discuss the convergence of factors related to race, pigmentation, and susceptibility to AMD, where the primary defect occurs in retinal support cells, the retinal pigment epithelium (RPE). We explore whether the observed racial bias in AMD incidence is related to innate differences in the basal level of pigmentation between races, and whether the pigmentation pathway activity in the RPE might protect from retinal degeneration. More specifically, we explore whether the downstream signaling activity of GPR143, a G-protein coupled receptor in the pigmentation pathway, might underly the racial bias of AMD and be a target to prevent the disease. Lastly, we summarize the past findings of a large retrospective study that investigated the relationship between the stimulation of GPR143 with L-DOPA, the pigmentation pathway, and AMD, to potentially help develop new ways to prevent or treat AMD. The reader of this review will come to understand the racial bias of AMD, which is related to the function of the RPE.

## 1. Introduction

**Figure 1:**
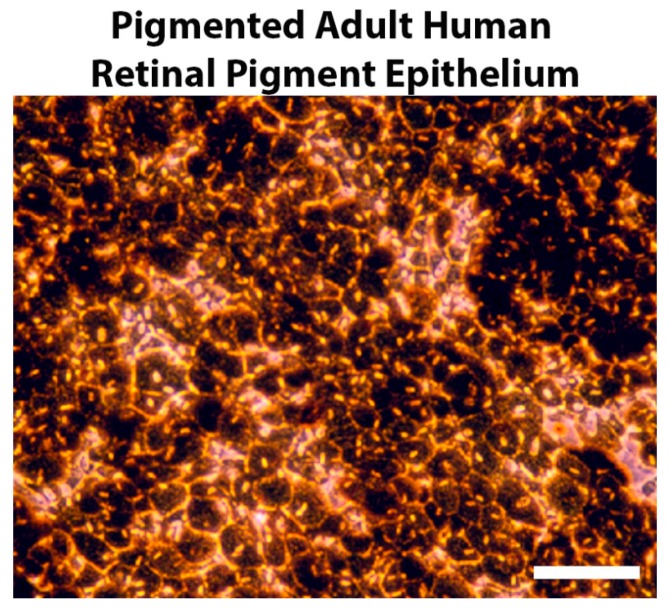
One of the many prominent features of the retinal pigment epithelium (RPE) is the production and accumulation of melanin granules. Representative phase-contrast micrograph of the cell morphology of human RPE cells from a 78-year-old healthy female donor. The primary cells were cultured with Dulbecco’s Modified Eagle Medium (DMEM) + 5% FBS. Total magnification 200×, scale bar = 25 µm.

The retinal pigment epithelium (RPE) is derived from the outer layer of the optic cup and consists of an epithelial monolayer located between the photoreceptor layer of the neural retina and the vascular bed of the choroid [1]. RPE cells are strongly polarized; they have a hexagonal-shape and house melanin granules that are apically distributed. Melanin granules appear early in development and decrease significantly in aging individuals, particularly in the macular RPE. Figure 1 illustrates the mosaic-like and pigmented single layer of RPE cells that were cultured from freshly enucleated human eyes by removing the anterior part of the globe, the vitreous, and retina, leaving behind an intact layer of RPE (methodology review: [2]). Primary RPE cultures typically show closely packed hexagonal cells that create a monolayer with varying degrees of pigmentation. Despite the homogeneous appearance of the RPE, the RPE monolayer in situ can be considered a mixture of heterogeneous cells that give rise to phenotypic differences when cultured [3]. For example, RPE cells may differ in levels of melanin accumulation in a regional pattern, with the lowest melanin content in the macula RPE and highest content in the far periphery [4]. However, others have suggested that different RPE regions have similar melanin content, but that choroidal melanin is greater in the macula and significantly greater in blacks than in whites [5]. These discrepancies may relate to the different methods used to quantify melanin. While these differences in the literature are important to acknowledge, our studies concentrate on the byproduct of melanin synthesis, L-dihydroxyphenylalanine (L-DOPA), which is a tyrosine metabolite that can cross all blood tissue barriers [6], such as the blood–ocular barrier and blood–brain barrier [7,8,9,10]. L-DOPA freely leaves cells and can easily cross tissue barriers, as indicated by its successful use to treat Parkinson’s disease, which requires L-DOPA to cross the blood–brain barrier and freely diffuse to enter neurons, where it is converted to dopamine [10,11]. At this point, the topographical distribution of melanin synthesis, the source of L-DOPA, is irrelevant, provided that it is in relative proximity. We hypothesize that the observed age-related melanin decrease relates to age-related macular degeneration (AMD) sensitivity, and it is due to reduced local L-DOPA and reduced pigmentation pathway signaling in the RPE.

Functionally, the RPE supports the outer sensory retina including the photoreceptors with transport activity between the retina and choroid, which is a typical function for an epithelium. However, the RPE supports photoreceptor function in multiple ways beyond simple nutrient transport; it absorbs scattered light, recycles retinal visual pigments, digests shed photoreceptor outer segments, and maintains the ionic balance in the subretinal space. Dysfunction of the RPE, and the associated retinal support, causes blinding diseases because the photoreceptors are dependent on the RPE. Thus, while the RPE cells do not directly participate in sensing light, they are vital to the development and maintenance of vision [1,12,13].

## 2. Pigmentation and Retinal Development

The RPE is the first tissue in the embryo to produce pigment during development, and failure of the RPE to produce pigment causes significant defects in retinal development [14,15,16]. Failure to synthesize melanin in the RPE, albinism, causes foveal hypoplasia, reduced numbers of photoreceptors and ganglion cells, and a reduction of the uncrossed optic projection at the optic chiasm, nystagmus, and strabismus [17]. Together, these neurosensory retina alterations cause low vision and reduced visual ability [18,19]. Albinism is a genetic disease and can affect eyes, hair, and skin, which is ocular cutaneous albinism (OCA), or just the eyes, which is ocular albinism (OA). OCA1–7 occur due to mutations in 7 genes with a variety of functions, including several enzymes and some small molecule transporters directly responsible for melanin synthesis (for a review, see [20]). OA is more specific and is caused by mutations in a single G-protein coupled receptor (GPCR) expressed in pigmented cells, GPR143. Interestingly, regardless of the genetic cause of albinism, OCA, or OA, the retinal defects are quite similar, suggesting that the underlying defect in RPE melanin accumulation is not proximal to the associated sensory retina alterations. The retinal neurons affected never express any of the genes linked to albinism, so a paracrine factor from the RPE is indicated [20]. While mechanistic defects in the ability to synthesize melanin are easy to grasp, for example in albinism caused by mutated tyrosinase, the downstream effects on retinal development are not obvious. Even more curious is that defects in GPR143 cause OA with the complete cadre of albinism-related retinal defects, despite normal or near normal pigmentation in the RPE. Thus, the OA phenotype demonstrates that the retinal changes associated with albinism are not tied to melanin accumulation itself; the actual production of melanin in the RPE appears unrelated to the visual defects in albinism. To make sense of this, let’s start with the following observations: the retinal albinism defects observed in OA are not tied to melanogenesis in the RPE; and the retinal defects associated with albinism must be paracrine in nature, because the retina does not express any pigmentation genes.

## 3. GPR143 and Pigmentation 

To address the role of RPE pigmentation during development, we concentrated our efforts on GPR143, because as a GPCR, it has the broadest ability to control RPE function. In addition, GPR143 sits at the most interesting place in the pigmentation pathway, where it separates RPE pigmentation from the hallmark albinism-associated retinal defects. We discovered that GPR143 is on the apical surface of human RPE in situ, rather than being intracellular as originally thought [21]. We also demonstrated that the endogenous ligand of the receptor is L-dihydroxyphenylalanine (L-DOPA). L-DOPA is a byproduct of the pigmentation pathway that is produced at the first step of melanin synthesis, when tyrosinase catalyzes the conversion of tyrosine to L-DOPA. All pigmented cells make and release L-DOPA. Thus, GPR143 signaling is directly related to pigment synthesis through tyrosinase activity and production of the ligand. The absolute similarity of the retinal defects from all types of albinism start to become clear as well. Each of the various mutations in different genes that cause albinism either lead to a reduction of tyrosinase activity and associated loss of L-DOPA production or loss of the receptor for L-DOPA, GPR143. From this point, we suggest that GPR143 signaling underlies a linkage between RPE pigmentation and support of the neurosensory retina.

A confounding variable, with respect to GPR143 signaling, is the accumulation of dopamine in the retina from the active signaling from retinal neurons that release dopamine during the day [22,23,24]. Thus, retinal dopamine exhibits a circadian rhythm, with the greatest levels of dopamine during the daylight hours. Dopamine is a direct competitive antagonist of GPR143 [21]. Of interest, L-DOPA and dopamine bind the same GPR143 binding site with similar affinity, but L-DOPA activates the receptor and dopamine inactivates it. Since dopamine inactivates the receptor and exhibits a circadian flux, the basal activity of GPR143 is likely to exhibit a circadian pattern. 

The source of L-DOPA that activates the receptor is uncertain. L-DOPA is produced by tyrosinase during melanin synthesis, and melanin synthesis occurs primarily during development [16]. Linking GPR143 signaling to retinal development is fairly simple; tyrosinase is active, and L-DOPA is produced and released to the subretinal space [16]. However, since melanin synthesis is minimal or absent in adults, the source of L-DOPA in the adult retina is not obvious. It is perhaps likely that a basal level of GPR143 activity exists in adults from either residual tyrosinase activity or spontaneous activation. Under such conditions, we would expect that the activity of GPR143 would likely be low, tied to basal ocular pigmentation, and circadian in nature due to competitive inhibition by dopamine.

## 4. Downstream Effectors of GPR143

As stated previously, there is evidence that RPE support for the neurosensory retina must be paracrine in nature, because the cells of the neural retina do not express the pigmentation pathway genes [20]. With respect to GPR143, this argument suggests that GPR143 signaling controls RPE paracrine release of something critical for retinal development and survival, because of the noted sensory retinal developmental defects when GPR143 is mutated, as in ocular albinism. To further investigate this line of reasoning, we sought to evaluate the RPE apical release of several putative agents with the potential to impact retinal development and biology. We found that GPR143 signaling increases the secretion of pigment epithelium-derived factor (PEDF) [21,25], reduces the secretion of vascular endothelial growth factor (VEGF) [25], and immediately reduces the apical release of exosomes [26]. PEDF and VEGF molecules have been extensively studied for their opposing effects in the control of tissue vascularity, where PEDF inhibits angiogenesis and VEGF promotes it [27,28]. To evaluate the changes in the expression of PEDF and VEGF in human RPE (hRPE) cells, enzyme-linked immunoabsorbent assays (ELISAs) were used to determine the concentration of both secreted proteins in the conditioned medium (CM) from stable monolayers of pigmented hRPE cells. We found that the regulation of both neurotrophic factor secretion is linked to an autocrine loop that regulates tyrosinase actity and melanin synthesis in the RPE, via the L-DOPA stimulation of GPR143. This finding was accomplished by inhibiting tyrosinase activity in pigmented RPE, which blocks the endogenous production of L-DOPA, the GPR143 agonist. Then, we determined the concentration of both secreted proteins in the CM from hRPE cells ± exogenous L-DOPA. Our results suggest that GPR143 signaling upregulates PEDF secretions and decreases VEGF secretion [21,25].

Beyond neurotrophic factor secretion, we also discovered that GPR143 signaling controlled exosome release [26]. Exosomes are derived from the endosomal compartment and are released when the multivesicular endosome (MVE), filled with intraluminal vesicles (exosomes when released), fuses with the plasma membrane. Exosomes were isolated by differential ultracentrifugation [29], and exosome quantitation was determined by both nanoparticle tracking analysis and total protein in CM collected from donor eyes after a 30 min incubation [26]. As such, the changes in exosome release are likely a direct reflection of the decreased rate of fusion of the MVE with the RPE plasma membrane or an increased rate of fusion of the late endosome with lysosomes. Our results illustrate that this aspect of RPE biology appears to be regulated by GPR143. The RPE factors that are altered are PEDF, VEGF, and exosome release, which may contribute to RPE protection from or participation in retinal disease.

### 4.1. Pigment Epithelium-Derived Factor

PEDF is a 50 kD secreted protein with known neurotrophic potential. PEDF was originally discovered based on the neurotrophic activity of conditioned medium from pigmented fetal human RPE cultures [30,31,32]. PEDF is structurally similar to a serpin, which is a family of serine protease inhibitors, however; PEDF does not demonstrate typical serpin-like activity. In PEDF knockout animals, it has been shown that PEDF likely serves as a negative control in retinal vascular development [33]. PEDF functions throughout the body, and it is likely active with the nuclear transcription factor nuclear factor kappa light chain enhancer of activated B cells (NF-κB). The protein is neuroprotective and neurotrophic, but at the same time it leads to apoptosis in vascular endothelial cells, illustrating that the effects of PEDF are cell-type specific [34]. Investigating RPE neurotrophic activity more broadly, we found that RPE promoted midbrain neuron survival and neurite out growth in a species-independent manner [35]. Using an unbiased screen, we showed that pigmented RPE fostered midbrain neurons and that most of the neurotrophic activity was through PEDF, corroborating the previously documented neurotrophic function of the protein [30,31,32]. In the RPE, GPR143 signaling in response to L-DOPA increases the secretion of PEDF.

### 4.2. Vascular Endothelial Growth Factor 

VEGF, a potent angiogenic factor, is a more complex group of proteins, related to platelet-derived growth factor (PDGF) [34]. Most studies involve the original VEGF, which is now identified as VEGF-A, however; VEGF-B, C, D, and placental growth factor (PIGF) also exist and have diverse functions. Further complicating the VEGF discussion is that VEGF-A has at least 7 isoforms produced by alternative splicing. These are tissue and context-specific, but generally speaking, VEGF causes vascular endothelial cell proliferation and new vessel formation and sprouting. The angiogenesis and cell proliferation activity of VEGF is through the stimulation of the VEGF receptor signaling, which is a family of receptor tyrosine kinases. Currently, the primary treatment for the neovascular form of AMD (wet AMD) is intravitreal injections of agents to block VEGF activity and halt angiogenesis. This strategy is effective, indicating that excessive VEGF activity is a component of wet AMD pathology [36,37,38]. GPR143 signaling in response to L-DOPA binding reduces the RPE secretion of VEGF.

### 4.3. Exosomes

**Figure 2:**
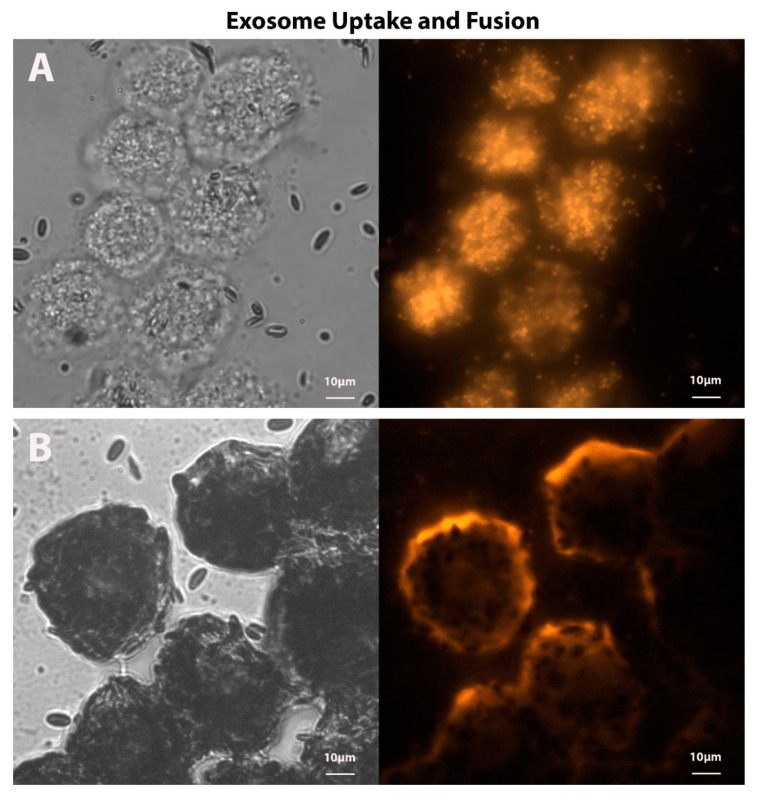
Representative images of possible mechanisms of intercellular communication by exosomes; exosome uptake mediated by endocytosis (**A**) or exosome fusion at the target cell’s plasma membrane (**B**). Paired phase-contrast and fluorescence micrographs of bovine ex-vivo RPE challenged with apical in situ bovine RPE exosomes. Exosomes were labeled with CF555 membrane protein dye to illustrate internalized exosomes taken up by the target unpigmented RPE cells from the tapetum lucidum region of the bovine eye (**A**) or lipophilic octadecyl rhodamine B, R18, membrane dye to illustrate sites of exosome fusion along the plasma membrane of pigmented RPE cells neighboring the tapetum lucidum (**B**). Total magnification = 630×.

Another downstream output of GPR143 signaling is the immediate halt to RPE exosome release [26]. The exact function of exosomes is not well understood, but exosomes may be a communication system between cells and tissues, and they may also initiate signaling through cell surface receptors [39,40,41]. Exosomes carry mRNA, miRNA, and bioactive functional proteins, all of which have the capability of altering the biologic activity of recipient cells. The delivery of exosome cargo can occur either by direct fusion of the exosome with the plasma membrane or by cellular uptake of the exosome. The delivery of exosome cargo can be visualized in the representative fluorescent micrographs of ex vivo RPE challenged with apical in situ RPE fluorescently-labeled exosomes (Figure 2). While the function of RPE exosomes with respect to activity in the retina or choroid remains elusive, the apical and basal RPE exosomes are distinct, indicating a significant level of cargo specificity [42,43]. Of interest with respect to GPR143 signaling is that L-DOPA stops exosome release in situ, suggesting that exosome release is controlled by at least one GPCR in the RPE—in this case, a GPCR tied to the pigmentation pathway. Significantly, this may well be the mechanism of action of L-DOPA in protection from AMD, since exosomes in other tissues and cancer cells drive angiogenesis through the miRNA cargo they carry [44,45]. In addition to a host of specific miRNA cargo, exosomes carry VEGF [46,47], which together can drive vascular cell proliferation and angiogenesis, indicating that exosomes may be stronger potentiators of angiogenesis than VEGF alone. Since the majority of AMD vison loss is related to angiogenesis, RPE exosomes seem to be a prime target to ameliorate AMD.

### 4.4. GPR143 Signaling 

One of the primary observations in the disease OA, caused by mutations in GPR143, is the formation of abnormal ‘macromelanosomes’ in the RPE and melanocytes [48,49,50]. Macromelanosomes occur through the abnormal growth of melanosomes and may represent a change in organelle motility and geography, in the absence of GPR143 signaling [51], due to the increase of improper transport vesicle fusion with the developing melanosome. Thus, it appears that during melanosome development, GPR143 signaling limits vesicle fusion. We also observed that GPR143 signaling rapidly stops exosome release, which may be analogous to GPR143 activity in melanosome maturation, where it limits vesicle fusion. Both exosomes and melanosomes are components of the endosomal compartment, and in both cases, GPR143 signaling appears to inhibit the membrane fusion of that compartment, but with different outcomes. In previous studies, we have shown that GPR143 signaling recruits the cytoplasmic protein myocilin to the endosomal compartment, where it is released from the cells on the surface of exosomes [29,52,53]. This observation provides direct evidence that GPR143 signaling causes cytoplasmic protein translocation to the exosome prior to release. At this time, it is unknown how GPR143 signaling may limit vesicle fusion, but the observation that it can change the recruitment of cytoplasmic protein to the vesicle surface opens a wide variety of possibilities, including the recruitment of Ras-related protein in the brain (RAB) proteins that could control fusion targets.

## 5. RPE Pigmentation in Aging

**Figure 3:**
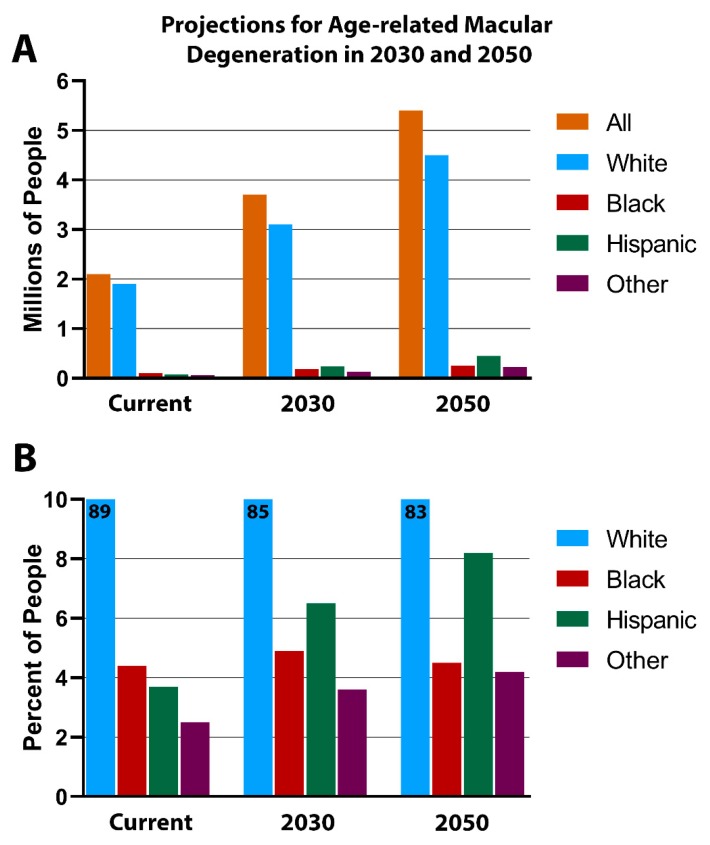
Projections for age-related macular degeneration. The white population will continue to account for most age-related macular degeneration (AMD)-related cases, as the disease incidence level is expected to more than double by 2050, from 2 million to 5.4 million people (**A**). The percentage of people affected with AMD by race is illustrated in (**B**). Races with the highest levels of pigmentation have the lowest incidence of AMD, which is possibly due to the stimulation of GPR143 by L-DOPA. Image adapted from the National Eye Institute’s AMD Data Tables, last updated 17 July 2019.

RPE pigmentation has a role in retinal health and survival well beyond development. The most common cause of blindness in the developed world is age-related macular degeneration (AMD) [54]. AMD is a racially biased disease, occurring much more commonly in those with the least pigmentation, the white population [55]. Life expectancy differs among the racial groups, which complicates our understanding of age-related diseases such as AMD. However, according to the Centers for Disease Control and Prevention (CDC) reports from 2014, Hispanic Americans’ life expectancy was 82, which is significantly longer than that of white Americans (79), while black and Native Americans both had shorter life expectancies (75 for both) [56]. Conversely, AMD incidence does not follow life expectancy differences among the races. For example, the white population has the greatest AMD incidence and reduced life span compared to the Hispanic American population, which has a longer life expectancy and simultaneously appears protected from AMD (Figure 3). As the American population ages, the number of those affected by AMD is increasing for all races, but this is not equivalent, as illustrated in Figure 3. Race, not life expectancy, is likely a crucial determinant of AMD incidence.

**Figure 4:**
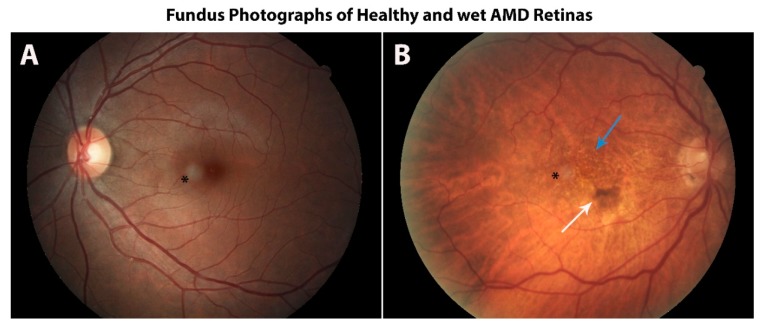
A healthy retina can undergo severe morphological changes due to wet age-related macular degeneration (AMD). Color photograph of a heavily pigmented fundus of a healthy volunteer (**A**) and the lightly pigmented fundus of a patient with wet AMD (**B**), which illustrates drusen (blue arrow) and retinal hemorrhage (white arrow). The black asterisk denotes a circular image artifact. (Images courtesy of Robert W. Snyder, MD, PhD).

The RPE is thought to be the primary tissue involved in AMD, but the pathobiology of the disease is uncertain. RPE perform many support functions for the outer retina, and the loss of this support likely underlies RPE participation in AMD. Of considered significance is that the RPE are post-proliferative; they do not divide and renew after early gestation [13]. The RPE are the most phagocytic and degradative tissue in the body, because every day, the end 10% of each photoreceptor is shed; then, it is engulfed and degraded by the RPE [1,57,58]. Each RPE cell is partnered with approximately 30 photoreceptors, so the daily phagocytic load is tremendous. With aging, the RPE develops signs of inefficient digestion, and it accumulates undigested proteins and lipids between the RPE and Bruch’s membrane called drusen; these acellular deposits can be found in the macular, perimacular, and peripheral retina and are heterogeneous in terms of shape, color, size, and content [59], 1980. Drusen and other anatomical changes may lie within normal signs of aging and can be observed during fundoscopic examination. Drusen can be categorized as hard or soft; hard drusen are smaller and have distinct margins, whereas soft drusen are larger with round indistinct margins [60]. In AMD, there are progressive macular disturbances of retinal pigmentation in addition to excess drusen formation, which can lead to RPE damage and a chronic aberrant inflammatory response that culminates in geographic atrophy, with the expression of angiogenic cytokines such as VEGF, or both. In the case of wet AMD, as illustrated in Figure 4, the fundus has both drusen and new blood vessels with exudate. With age, the macular RPE cells also lose 30% of their melanin content [13], so simultaneously RPE cells are being stressed by the phagocytic load, and the pigment associated with protection is declining. GPR143 signaling activity appears to be directly linked to the level of pigmentation, suggesting that GPR143 activity is declining in the RPE with age.

## 6. L-DOPA and AMD 

We developed the hypothesis that RPE pigmentation, inextricably linked to GPR143 signaling activity, protects from AMD. The ligand for GRP143 is L-DOPA, which is a common pharmaceutical that is used to treat movement disorders such as Parkinson’s disease (PD) [10,61]. PD incidence increases with age, and the PD patient population overlaps those at risk from AMD. With this knowledge, a retrospective analysis was conducted to evaluate whether individuals taking L-DOPA, largely for PD, were protected from AMD. The study investigated several non-overlapping electronic medical records and claims databases using International Statistical Classification of Diseases and Related Health Problems (ICD-9) codes for AMD diagnoses (includes both wet and dry AMD) and L-DOPA prescriptions from each database [62]. The three databases used for the retrospective study were the clinical data from the Marshfield Clinic’s Personalized Medicine Research Project (20,000 individuals), electronic health records from a non-overlapping group from Marshfield Epidemiologic Study Area (17,500 individuals), and a claim-based Truven MarketScan outpatient database (15,215,458 individuals). The study showed that the age of L-DOPA prescription was similar in the three databases (67.1, 67.2, 68) and likewise, the age of AMD diagnoses for those without an L-DOPA prescription were nearly identical (71.2, 71.3, 71.4) and matched the expected AMD age of diagnosis [63]. However, the results illustrated an intersection between L-DOPA prescription and age of AMD onset, suggesting that L-DOPA protected from AMD. The diagnosis of AMD in patients with a L-DOPA prescription increased from 71 to 79.3 years old, and similar results were shown for wet AMD, delaying onset from 75.8 to 80.8 years of age. Since the onset of wet AMD follows the existing and ongoing dry AMD pathogenesis, the delayed age of onset observed for wet AMD suggests there may be a stabilization of AMD pathology in patients that were taking L-DOPA. Similarly, L-DOPA significantly reduced the probability of ever being diagnosed with either form of AMD in aging populations. Collectively, the retrospective data strongly suggest that L-DOPA may prevent any type of AMD, stabilize dry AMD, and delay the onset of both dry and wet AMD. By extension, since GPR143 is the only known L-DOPA receptor, we suggest that GPR143 activity may protect from the disease. This suggestion is strongly supported by the racial bias of AMD incidence discussed above and the fact that the activity of the receptor is directly linked to the accumulation of melanin.

GPR143 signaling by L-DOPA reduces VEGF secretion, increases PEDF secretion, and immediately decreases exosome release. The alteration in VEGF and PEDF secretion are significant and reproducible, but modest. The effect of GPR143 signaling on exosome release was immediate and dramatic. How might stopping exosomes help with AMD?

### Exosomes, GPR143, and AMD

- AMD is caused, or accompanied by diminished choroidal perfusion and RPE hypoxia.
- Hypoxia causes cells to release exosomes.
- Exosomes stimulate angiogenesis.
- Retinal neovascularization is a primary cause of vision loss in AMD.
- The ligand of GPR143 is L-DOPA.
- L-DOPA stops RPE exosome release.
- L-DOPA systemically treats wet AMD.

Together, our line of resoning incorporates the natural incidence and pathobiology of AMD; the expected universal response of cells to hypoxia, exosome release; and our data that GPR143 signaling stops exosome release. We hypothesize that exosomes are a significant component of AMD pathology, and that GRP143 can be an ideal pharmacological target to treat the disease.

In response to our retrospective study, four clinical trials have been developed to prospectively test whether L-DOPA can treat AMD. The first was conducted in France, Fondation Ophtalmologique Adolphe de Rothschild (ClinicalTrials.gov identifier, NCT03415984), which was designed around AMD incidence in elderly PD patients taking ± L-DOPA for therapy. In both groups, AMD was diagnosed based on three exams: color retinography, optical coherence tomography (OCT), and fundus autofluorescence imaging. The study has been completed, but the results have not been published. Three other trials have been designed by our clinical collaborators and are ongoing. A one-month study (ClinicalTrials.gov identifier, NCT03022318) was designed to determine whether L-DOPA can delay anti-VEGF injection therapy in newly diagnosed patients with wet AMD that are naïve to anti-VEGF injections. The second and related third clinical trials, (ClinicalTrials.gov identifier, NCT03023059 and NCT03197493) are both dose-ranging studies for L-DOPA effects on wet AMD. These studies are all ongoing with expected completion dates of February 2020 (2318), February 2021 (3058), and May 2021 (7493). The results for these three studies have not been released. One of the known therapeutic effects of intravitreal anti-VEGF injection therapy is the reduction of retinal fluid accumulation from blood vessels that leak fluid and blood. The activity of VEGF leads to the ingrowth of abnormal blood vesels from the choriocapillaris into the subretinal or sub-pigment epithelial space, which can cause retinal fluid accumulation, such that reducing RPE secretion of VEGF by the stimulation of GPR143 with L-DOPA may reduce retinal fluid accumulation. It will be interesting to evaluate whether L-DOPA treatment of patients with wet AMD, which is expected to reduce VEGF secretion from RPE, also reduces retinal fluid accumulation.

Given the likely circadian nature of GPR143 activity, clinical trials to investigate L-DOPA for AMD should take into account when patients take the medicine. As previously mentioned, in the morning and during the day, the retina produces and releases the antagonist of GPR143, dopamine. In contrast, at night, retinal dopamine is lower. To get the most effect out of the stimulation of GPR143 activity with L-DOPA, perhaps the medicine should be taken before bedtime to increase effectiveness.

## 7. Conclusions 

**Figure 5:**
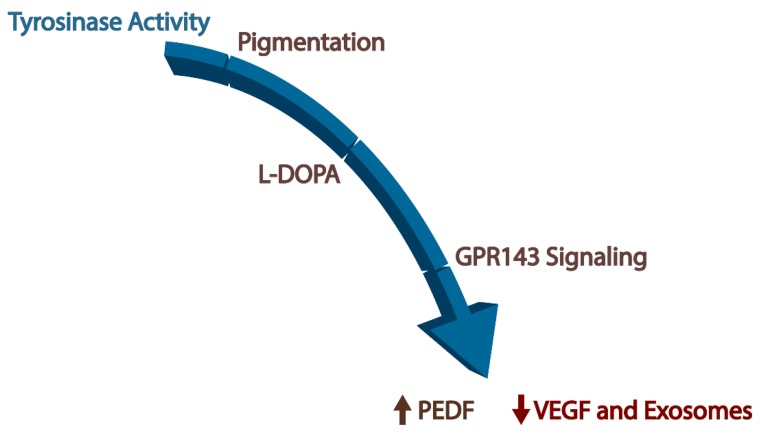
Schematic diagram of the path from tyrosinase activity in the retinal pigment epithelium to increased pigment epithelium-derived factor (PEDF) secretion, decreased secretion of vascular endothelial growth factor (VEGF), and decreased release of exosomes. Together, these downstream effectors of GPR143 signaling by L-DOPA are likely to offer protection of the retina during aging.

Albinism is a group of rare genetic disorders typically associated with a defect in melanin accumulation. In another form of albinism, ocular albinism (OA), pigmentation is normal, but the same retinal defects occur, separating pigmentation from retinal development. Can the visual deficits reported in people with albinism be rescued by stimulating a known participant of the pigmentation pathway, GPR143? Studies in a mouse model of human albinism report that early post-natal supplementation with L-DOPA significantly recovers retinal development only if L-DOPA is administered in a known period of retinal plasticity [64]. In contrast, a similar study in young adults with albinism demonstrated that L-DOPA did not have a beneficial effect, perhaps owing to the developmental origin of the retinal changes [65]. In the case of AMD, which is the most common cause of blindness in the developed world with incidence and vision loss displaying a strong racial bias, we developed the hypothesis that the racial bias observed in those protected from AMD occurs as a result of increased pigmentation pathway activity in the RPE. While investigating this idea, we discovered the ligand (L-DOPA) for a key GPCR in the pigmentation pathway, GPR143. We analyzed the downstream outputs from the GPR143 signaling pathway that may relate to the racial or pigmentation bias of the disease (illustrated in Figure 5), and then we tested whether supplementation with the ligand for GPR143 treats AMD. In addition to L-DOPA, GPR143 responds to dopamine, which is a neurotransmitter that plays a role in the entrainment of RPE circadian clocks [66]. Dopamine is released by retinal neurons during daylight hours and inactivates GPR143, unlike L-DOPA, which activates GPR143. The signaling of GPR143 appears to respond to dopamine and L-DOPA in a circadian fashion and needs further exploration to better understandand the role of GPR143 in rescuing AMD pathogenesis.

In summary, this review has primarily focused on blinding diseases that are characterized by:

1. The absence of GPR143 signaling/disruption of the pigmentation pathway, as observed in ocular albinism (OA).
2. Race and age-related decrease of GPR143 signaling, as observed in the prevalence of age-related macular degeneration (AMD) in whites compared to other races.

Curiously enough, despite the high prevalence of AMD in people over the age of 65, clinicians have rarely identified a patient with both albinism and AMD. Are people with albinism protected from AMD? This is a question worth exploring that may aid in further understanding the link between the pigmentation pathway and AMD.
